# A Mighty “Protein Extractor” of the Cell: Structure and Function of the p97/CDC48 ATPase

**DOI:** 10.3389/fmolb.2017.00039

**Published:** 2017-06-13

**Authors:** Yihong Ye, Wai Kwan Tang, Ting Zhang, Di Xia

**Affiliations:** ^1^Laboratory of Molecular Biology, National Institute of Diabetes and Digestive and Kidney Diseases, National Institutes of HealthBethesda, MD, United States; ^2^Laboratory of Cell Biology, Center for Cancer Research, National Cancer Institute, National Institutes of HealthBethesda, MD, United States

**Keywords:** AAA ATPase, p97/VCP, Cdc48, chaperones, protein denaturation, protein quality control, neurodegenerative diseases

## Abstract

p97/VCP (known as Cdc48 in *S. cerevisiae* or TER94 in *Drosophila*) is one of the most abundant cytosolic ATPases. It is highly conserved from archaebacteria to eukaryotes. In conjunction with a large number of cofactors and adaptors, it couples ATP hydrolysis to segregation of polypeptides from immobile cellular structures such as protein assemblies, membranes, ribosome, and chromatin. This often results in proteasomal degradation of extracted polypeptides. Given the diversity of p97 substrates, this “segregase” activity has profound influence on cellular physiology ranging from protein homeostasis to DNA lesion sensing, and mutations in p97 have been linked to several human diseases. Here we summarize our current understanding of the structure and function of this important cellular machinery and discuss the relevant clinical implications.

p97/Cdc48 belongs to the AAA+ (extended family of ATPases associated with various cellular activities) ATPase family, which functions generally as essential chaperones to promote protein folding or unfolding. Cdc48 was initially identified in *S. cerevisiae* as a cell cycle regulator, which upon inactivation, leads to a cell cycle arrest at the G2-M transition stage (Moir et al., 1982). A mammalian homolog of 97 kDa was later discovered and dubbed as p97 or valosin-containing protein precursor (VCP) (Koller and Brownstein, 1987). In *Drosophila*, the name TER ATPase (transitional endoplasmic reticulum ATPase) has been used given the partial localization of this enzyme to the endoplasmic reticulum (ER) surface (Zhang et al., 1994, see below). In this review, we use p97 and Cdc48 to refer to the mammalian and yeast homologs, respectively.

As a type II AAA+ ATPase, p97/Cdc48 has two AAA ATPase domains designated as D1 and D2 (Figure 1A). These two domains are connected by a short polypeptide linker (D1–D2 linker). Although the ATPase domains are highly similar in sequence and structure, they have distinct functions: while the D1 domain is required for hexameric assembly of p97, the D2 domain is a major contributor of the overall ATPase activity (see below, Song et al., 2003; Wang et al., 2003). In addition, p97/Cdc48 has a sizable N-terminal domain (N-domain) that is linked to the D1 domain by a flexible polypeptide segment (N-D1 linker). At the C-terminus, a short tail is appended to the D2 domain. The interaction of p97/Cdc48 with its partners is mostly mediated by the N-domain, but a few proteins bind p97/Cdc48 using its C-terminal tail (Ogura and Wilkinson, 2001; Buchberger et al., 2015).

**Figure 1:**
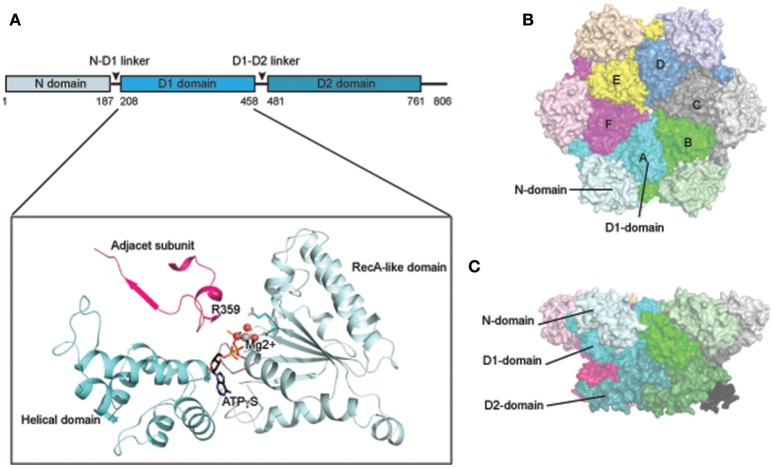
Structure of p97/Cdc48. **(A)** Cartoon representation of the domain organization of p97/Cdc48. Color code reflects that for subunit A in **(B,C)**. The ribbon structure shows the D1 domain of a single protomer bound by a ATPγS molecule (PDB:4KO8). The RecA-like domain is colored in light blue and the characteristic helical domain is in cyan. The nucleotide-binding site communicates with a neighboring subunit through the SRH (second region of homology, in red) motif, where a conserved Arg-finger residue R359 is in contact with the bound nucleotide. **(B,C)** Surface representation of the structure of hexameric p97 (PDB: 3CF2 in the ADP-bound form) **(B)** is a top view down the 6-fold symmetry axis showing the N-D1 ring. The six subunits are labeled in colors. The D1 domain and the N-domain are indicated with arrows and labeled for one of the six subunits. **(C)** is a side view of the p97 hexamer.

As a soluble protein, p97 is primarily localized in the cytosol, but a fraction is present on organelle membranes including the endoplasmic reticulum (ER), Golgi, mitochondria, and endosomes (Acharya et al., 1995; Latterich et al., 1995; Rabouille et al., 1995; Xu et al., 2011; Ramanathan and Ye, 2012). How p97/Cdc48 is recruited to different membranes is largely unclear, but this process is probably mediated by adaptors on different organelles, as demonstrated for the ER (Christianson and Ye, 2014). A fraction of p97/Cdc48 is also localized in the nucleus (Madeo et al., 1998), where it assists various chromatin-associated processes or nuclear protein quality control (PQC) (see below).

In multicellular organisms, the expression of p97 is ubiquitous. In humans, the transcription of p97 was moderately upregulated in some cancers, and the level of p97 mRNA appears to correlate with cell sensitivity to cell death induced by a potent p97 inhibitor, a potential anti-cancer drug (Anderson et al., 2015). More recently, genetic studies revealed that mutations in p97 may be causal to several human diseases including IBMPFD (Inclusion Body Myopathy associated with Paget's disease of the bone and Frontotemporal Dementia) and amyotrophic lateral sclerosis (ALS) (Xia et al., 2016). These findings stimulated a flurry of investigations on p97 substrates whose “mis-handling” by p97 mutants may have caused abnormality in human physiology.

Most p97/Cdc48 substrates identified to date are conjugated with ubiquitin and targeted for degradation by the 26S proteasome, but a few exceptions exist (Ramadan et al., 2007; Wilcox and Laney, 2009; Ndoja et al., 2014). A key feature of the p97/Cdc48-assisted degradation system is that many cofactors or adaptors are capable of recognizing ubiquitin conjugates (Ye, 2006). Some p97 cofactors are enzymes that can add or remove ubiquitin conjugates, but most of them, regardless of whether or not possessing a ubiquitin binding motif, seem to serve an adaptor function that links this ATPase to a specific subcellular compartment or substrate.

## Structure of p97

p97 forms a stable hexameric structure with two concentric rings (Figures 1B,C): the N-D1 ring has the N-domains laterally attached and therefore has a larger radius (Peters et al., 1990; Zhang et al., 2000; DeLaBarre and Brunger, 2003, 2005; Huyton et al., 2003; Davies et al., 2008; Banerjee et al., 2016; Schuller et al., 2016). A similar ring-shaped structure was observed for various IBMPFD mutants (Tang et al., 2010; Tang and Xia, 2012, 2013) and for wild-type p97 that is in complex with cofactors or adaptors (Dreveny et al., 2004; Ewens et al., 2014; Hanzelmann and Schindelin, 2016a). The hexameric assembly of p97 is dependent on the D1 domain, but is stable in the absence of nucleotide (Wang et al., 2003).

As in all AAA+ ATPases, the AAA module of p97/Cdc48 consists of a characteristic helical domain and a highly conserved RecA-like domain (Figure 1A). The RecA-like domain features a nucleotide-binding site at the interface between two adjacent subunits. In this configuration, arginine-finger residues (R359 and R635 for the D1 and D2 ring, respectively) can promote nucleotide hydrolysis by engaging the γ-phosphate of ATP that is bound to an adjacent subunit. In addition, the active site contains a Walker A [P-loop, G(x)4GKT, x is any residue] motif for nucleotide binding and Walker B motif (hhhhDE, h represents hydrophobic residues) for nucleotide hydrolysis (Ogura and Wilkinson, 2001).

## Nucleotide binding and hydrolysis

Purified p97 hydrolyzes 1–5 ATP molecules per hexamer per second *in vitro* (Meyer et al., 1998; Song et al., 2003; Ye et al., 2003; Tang and Xia, 2013). The ATPase activity of p97 can be influenced by physical parameters such as temperature, the position of the N-domain, and adaptor (Meyer et al., 1998; Song et al., 2003; DeLaBarre et al., 2006; Niwa et al., 2012; Zhang X. et al., 2015; Bulfer et al., 2016). Importantly, two recent reports showed that the ATPase activity of p97 and CDC48 can be activated moderately by a ubiquitinated model substrate (Blythe et al., 2017; Bodnar and Rapoport, 2017), consistent with genetic studies demonstrating that ATP hydrolysis is indispensable for all documented p97 functions (Kobayashi et al., 2002; Ye et al., 2003; Dalal et al., 2004; Raman et al., 2011; Xu et al., 2011, 2016).

Nucleotides binding to p97 has been measured by isothermal titration calorimetry (ITC) (Briggs et al., 2008; Tang et al., 2010) or by surface plasmon resonance (SPR) (Chou et al., 2014). Although there is a 10-fold difference in measured affinities, the relative affinity of D1 and D2 to nucleotide is comparable between these methods. For isolated wild-type p97, the D1 and D2 domains bind ADP with K_d_ of ~1 μM and ~80 μM, respectively, but the affinity for ATP and ATPγS is about the same (~2 μM) for these domains (Briggs et al., 2008). A remarkable observation, though not yet fully appreciated, is the existence of pre-bound or occluded ADP in the D1 domains, which may regulate the asymmetric movement of the N-domain (Tang et al., 2010; Tang and Xia, 2016a). Davies and colleagues first reported using chemical denaturation experiments that about half of the D1 sites in wild-type p97 hexamers are pre-occupied by ADP (Davies et al., 2005). It was subsequently shown that the D1-bound ADP molecules are difficult to remove *in vitro*, raising concerns about interpreting results from various *in vitro* ATP binding and hydrolysis experiments (Briggs et al., 2008; Tang et al., 2010).

*In vitro* studies showed that the two ATPase domains of p97 are not functionally equivalent, as the D2 domain reportedly displays a higher ATPase activity than D1 (Song et al., 2003). Whether the D1 and D2 rings work independently or communicate with each other during the ATP hydrolysis cycle has been studied extensively, though the results reported are not always consistent. By measuring the activity of each ring while inhibiting the other, an early report suggested that the two ATPase rings operate independently (Song et al., 2003), but others showed evidence of inter-ring communications (Beuron et al., 2003; Ye et al., 2003; Chou et al., 2014). Moreover, intricate allosteric communication between ATPase domains within the same ring has been suggested (Nishikori et al., 2011; Hanzelmann and Schindelin, 2016b). These interactions are thought to coordinate domain movement during the ATP hydrolysis cycle.

## Nucleotide-dependent conformational changes

The conformational dynamics of p97 has been elusive, in part owing to difficulties in studying its structure under physiologically relevant *in vitro* conditions. The issue is further complicated by the occluded D1 nucleotide, which excludes other nucleotides from the same site. Furthermore, structural studies by crystallography often require proteins in different asymmetric units to take a similar conformation, but the six ATPase domains are not synchronized in nucleotide binding and hydrolysis. Despite of these challenges, conformational changes of p97 have been intensively pursued by both cryo-EM and X-ray crystallography. Early cryo-EM studies revealed moderate rotational movement between the two ATPase rings upon ATP hydrolysis as well as closure and opening of the D1 or D2 central channel (Rouiller et al., 2002). Other domain movements were also noted (Beuron et al., 2003). However, due to limited resolution, these studies failed to generate a consistent model. The issue was revisited more recently with the application of newer technologies. One study using high-speed atomic force microscopy showed a conformational change in CDC48.1, a *C. elegans* p97 homolog, which involves rotation of the ND1 ring back and forth relative to the D2 ring following D2 ATP hydrolysis (Noi et al., 2013). Likewise, another study by single-particle Cryo-EM reported two nucleotide dependent conformations, differentiated by inter-ring rotation of approximately 22° (Yeung et al., 2014).

Crystallographic studies initially suggested that nucleotide-dependent conformational changes might take place only during the D2 ATP hydrolysis cycle because D1 appeared to be constantly occupied by ADP (Zhang et al., 2000; DeLaBarre and Brunger, 2003, 2005; Huyton et al., 2003; Davies et al., 2008). To date, the most significant structural change associated with the D2 ATPase cycle is the opening of the D2 pore and an inter-ring rotation mentioned above, but whether the D2 pore opening is triggered by nucleotide binding or hydrolysis is unclear (Rouiller et al., 2002; Davies et al., 2005, 2008; Pye et al., 2006; Banerjee et al., 2016; Hanzelmann and Schindelin, 2016b; Schuller et al., 2016). Additionally, part of the D2 domain also undergo an order-to-disorder transition (DeLaBarre and Brunger, 2005).

It has only become clear recently that the D1 domain in p97 can also hydrolyze ATP under physiological conditions. Studies using D2 specific p97 ATPase inhibitor demonstrated that the D1 domain contributes significantly (~30%) to the overall ATPase activity (Chou et al., 2014; Anderson et al., 2015). Because genetic evidence showed that certain *Cdc48* D1 mutants cannot rescue the growth defect of Cdc48 temperature sensitive alleles despite carrying an intact D2 domain, the D1 domain clearly has an important function (Ye et al., 2003; Nishikori et al., 2011).

**Figure 2:**
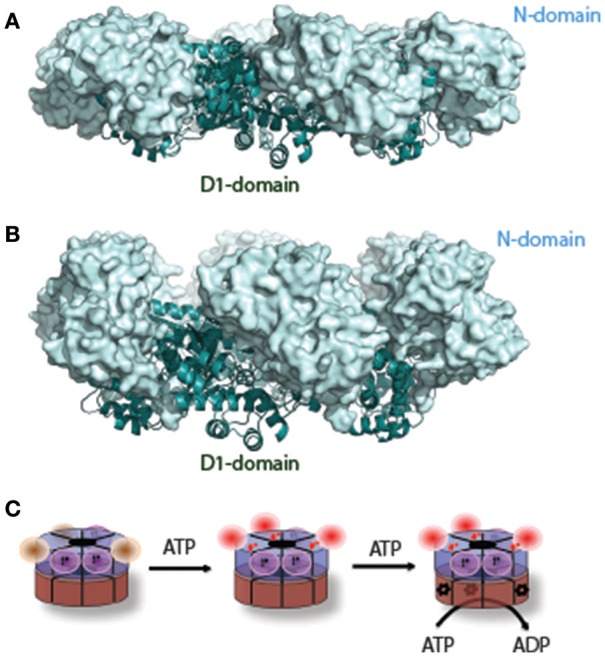
A nucleotide-dependent N-domain conformational change. **(A)** When ADP is bound to the D1 domain in ribbon diagram in cyan, the N-domain (in light-blue surface representation) assumes the Down-conformation (PDB: 1E32, wild type N-D1). **(B)** When ATP is bound to the D1 domain, the N-domain moves to the Up-conformation (PDB: 4KO8, R155H mutant N-D1). **(C)** A schematic model of the N-D1 conformational change upon D1 ATP hydrolysis. The p97 hexamer is represented as two concentric rings with D1 in blue and D2 in brown. The N-domains in the Down-conformation are shown as magenta balls and their cognate D1 domains are occupied with occluded ADP (labeled D). D1 domains with empty nucleotide-binding pockets are not labeled and their cognate N-domains are likely to be mobile (brown balls). ATP binding to the empty sites of the D1 domains will lead the N-domains to the Up-conformation. Occupation of ATP to the D1 domain renders the cognate D2 domain capable of hydrolyzing ATP, which is labeled with a red ^*^. The D1 domain probably hydrolyzes ATP once a few D2 domains have been converted to the ADP bound state.

Whether ATP hydrolysis by D1 is essential for p97 function has been a controversial issue. Nevertheless, D1-dependent conformational changes have been extensively sought by various biophysical approaches and were recently reported by several groups. Retrospectively, a major obstacle in studying D1-dependent conformational changes was the presence of sub-stoichiometric amount of tightly bound ADP in the D1 nucleotide-binding site (Davies et al., 2005; Tang and Xia, 2013). One strategy to circumvent this problem in crystallographic study is to use p97 mutant proteins bearing amino acid substitutes found in IBMPFD (Inclusion Body Myopathy associated with Paget's disease of the bone and Frontotemporal Dementia syndrome) patients (Kimonis et al., 2000). When purified, the D1 domain in these mutants can efficiently bind to exogenously added nucleotides, allowing crystallographic studies of conformational changes that occur during the D1 ATPase cycle. Strikingly, compared to structures in which D1 is in the ADP-bound state (Down-conformation, Figure 2A), in the presence of the ATP analog ATPγS in D1, the N-domain undergoes a hinged upswing (Up-conformation, Figure 2B) (Tang et al., 2010; Xia et al., 2016). A similar conformational change was seen with wild-type p97 in solution by small-angle X-ray scattering (SAXS) (Tang et al., 2010). As it turns out that the difference between wild-type and mutant p97 lies in that for p97 mutant all six N-domains undergo a uniform conformational change, allowing X-ray crystallographic studies, whereas for wild-type p97 only a fraction of the six subunits have the N-domains in the Up-conformation (Tang and Xia, 2016a). Thus, unsynchronized nucleotide binding and hydrolysis seems to be a common feature for both D1 and D2, which might be functionally relevant to the observed asymmetric adaptor-binding to the p97 N-domain (Buchberger et al., 2015).

The above-mentioned conformational changes in the N-domain were lately confirmed by cryo-EM studies. One study found p97 in three different, co-existing states in the presence of ATPγS in solution: one has ADP bound to all 12 sites and the N-domains in the Down conformation; the second, also in the Down conformation, has the six sites in the D1-ring and the six sites of the D2-ring occupied by ADP and ATPγS, respectively; in the third case, all 12 sites contain ATPγS and now the N-domains are held in the Up-conformation (Banerjee et al., 2016). It should be noted that while the EM densities for the D1 and D2 domains are well defined, those for the N domains are not, particularly for the one with full occupancy of ATPγS. The poor density for the N-domains suggests disorder or multiple conformations. Indeed, in another study, carefully sorted images of wild-type p97 prepared in the presence of AMP-PNP showed that even different protomers within a single hexameric p97 molecule display significant asymmetric domain movement, resulting in a random distribution between the Up- and Down-conformations in solution (Schuller et al., 2016). The nucleotide-dependent Up and Down conformational switch of the N domain in the context of the N-D1 fragment was also confirmed recently by NMR (Schuetz and Kay, 2016).

## Mechanism of force generation

A major unresolved issue in the field is how conformational changes in p97 generate the proposed “segregase” activity. To date, the most consistent conformational changes observed are the D2 rotation-accompanied pore opening/closing and the up-and-down swing motion of the N-domain. While the former appears to be linked to the D2 ATPase cycle, the latter is driven entirely by nucleotide hydrolysis in the D1 domain (Figure 2C). Force generation presumably requires cooperation between the D1 and D2 rings, which would explain the observed interdomain communications (Beuron et al., 2003; Ye et al., 2003; Chou et al., 2014; Schuetz and Kay, 2016).

The force applied onto a substrate may result in partial unfolding of a client protein, and thus disrupt its interaction with protein assemblies, membranes, or chromatin. Although many AAA+ proteins are protein unfoldase (e.g., ClpA and ClpX) that threads polypeptides through a central tunnel (Singh et al., 2000), p97 cannot unfold GFP-ssrA, a model aberrant substrate (Rothballer et al., 2007). By contrast, VAT, a *thermoplasma acidophilum* p97 homolog, is capable of unfolding GFP-ssrA with a low efficiency (Gerega et al., 2005). Intriguingly, this unfolding activity can be dramatically enhanced when the N-domain of VAT is deleted (Gerega et al., 2005; Barthelme and Sauer, 2012). N-deleted VAT can also collaborate with the 20S proteasome to degrade GFP-ssrA *in vitro* (Barthelme and Sauer, 2016). Protein sequence analyses identified a KYYG motif in a D1 loop of VAT, which is replaced by KLAG in p97. When these tyrosine residues are introduced to replace leucine or alanine in a p97 variant lacking the N domains, it now can unfold and target GFP-ssrA to the 20S proteasome for degradation (Rothballer et al., 2007; Barthelme and Sauer, 2013). Collectively, these findings indicate that the widely observed cooperation between AAA+ ATPases and the 20S proteasome is an ancient scheme of protein degradation. However, with evolved changes in the N-domain and the D1 ring, p97 appears to acquire a more sophisticated mechanism to process its substrate. It has been speculated that p97/CDC48 might function as a special “unfoldase,” perhaps only with the assistance from ubiquitin molecules conjugated to its substrate. Consistent with this view, the requirement of p97/Cdc48 in protein degradation *in vivo* can be bypassed if a flexible peptide was fused to the C-terminus of a proteasome substrate (Beskow et al., 2009), suggesting that p97/Cdc48 may initiate protein unfolding to expose a loosely-folded segment for subsequent engagement of the proteasome. More direct proof of the ubiquitin dependent unfoldase hypothesis came from two recent studies (Blythe et al., 2017; Bodnar and Rapoport, 2017), which used *in vitro* reconstitution systems to show that both p97 and its yeast homolog CDC48 can unfold GFP, but only when it carries ubiquitin conjugates. As expected, this activity is dependent on the D2 ATPase activity, the cofactors Ufd1 and Npl4, and on the length of the ubiquitin chains on GFP. Intriguingly, the D1 ATP hydrolysis does not seem to contribute significantly to GFP unfolding in a single round GFP turnover assay (Barthelme and Sauer, 2013). However, it appears to be required for substrate release from CDC48 to ensure processivity. Importantly, the study by Bodnar and Rapoport demonstrates, using two polyubiquitinated model substrates, that once ubiquitin chains are partially trimmed substrates can be completely threaded through the central pore of p97 together with the remaining ubiquitin molecules in a D1 to D2 direction, which results in unfolding of these proteins. The ubiquitin trimming reaction is dependent on an intricate interplay between p97 and its associated deubiquitinase Otu1 (Bodnar and Rapoport, 2017).

## p97-interacting proteins

**Figure 3:**
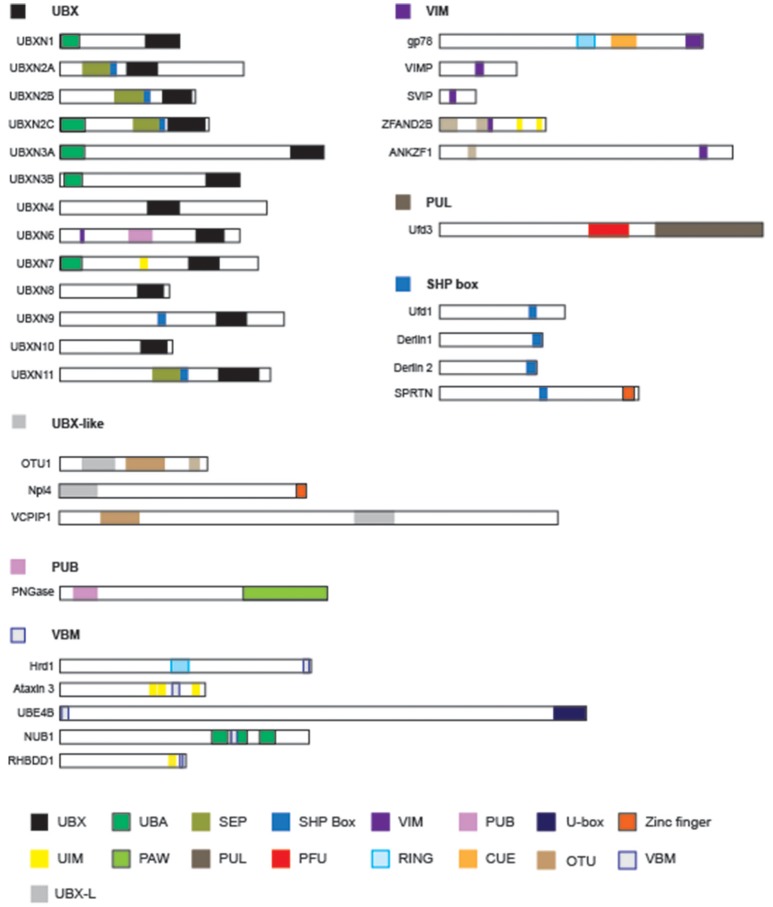
The domain structures of p97-interacting proteins. Known p97-interacting proteins are grouped by their p97-interacting domains, which are highlighted by colored boxes: UBX, ubiquitin like/ubiquitin regulatory X; UBA, ubiquitin associated; SEP, Shp1, Eyc and p47; VIM, VCP interacting motif; PUB, Peptide:N-glycanase/UBA or UBX-containing proteins; UIM, ubiquitin interacting motif; PAW, domain present in PNGases and other worm proteins; PUL (PLAP, UFD3 and Lub1) domain; PFU, PLAA family ubiquitin binding; RING, really interesting new gene; CUE (Coupling of Ubiquitin to ER-degradation) domain; UIM, ubiquitin interacting motif. The schematic representations are drawn to scale.

Proteomic studies have identified many factors that interact with p97/Cdc48 (Alexandru et al., 2008; Buchberger et al., 2015; Raman et al., 2015). These factors can be categorized either as adaptors, which link p97/Cdc48 to a specific substrate in a subcellular compartment, or as cofactors that facilitate substrate processing. Cofactors usually have enzymatic activities [e.g., N-glycanase, ubiquitin ligase, or deubiquitinase (DUB)] that can alter protein modifiers present on substrates (Figure 3).

Some p97/Cdc48-interacting proteins including PLAA/Ufd3, PNGase, HOIP, and Ufd2 bind to the C-terminal appendage of p97/Cdc48 (Rumpf and Jentsch, 2006; Zhao et al., 2007; Qiu et al., 2010; Bohm et al., 2011; Schaeffer et al., 2014; Murayama et al., 2015), but the vast majority bind p97/Cdc48 through its N-domain (Table 1) (Buchberger et al., 2015). Sequence analyses have revealed several p97-interacting patterns including VIM (VCP-interacting motif) (Stapf et al., 2011), UBX (ubiquitin regulatory X) (Buchberger et al., 2001; Schuberth and Buchberger, 2008), VBM (VCP-binding motif) (Boeddrich et al., 2006), and SHP box (also known as binding site 1, bs1) (Bruderer et al., 2004). The VCP-interacting motif (VIM) is a linear sequence motif (RX_5_AAX_2_R) present in gp78 (Ballar et al., 2006), SVIP (small VCP-inhibiting protein) (Ballar et al., 2007), VIMP (VCP-interacting membrane protein) (Ye Y. et al., 2004), VMS1 (Heo et al., 2010), UBXN6 (Hanzelmann and Schindelin, 2011; Stapf et al., 2011), and ZFAND2B (Stanhill et al., 2006). By contrast, the VBM domain found in proteins such as ataxin-3, Ufd2 and Hrd1 features a polarized sequence motif (RRRRXXYY) (Boeddrich et al., 2006). The SHP box in p47 (Kondo et al., 1997), Ufd1 (Meyer et al., 2000), and Derlin-1 (Lilley and Ploegh, 2004; Ye Y. et al., 2004; Greenblatt et al., 2011) on the other hand is a short polypeptide segment enriched in hydrophobic residues. Noticeably, the UBX domain, an 80-residue module structurally related to ubiquitin, is present in a p97/CDC48 adaptor family known as UBX-containing proteins, consisting of 13 members in humans (Table 1).

**Table 1:** p97-interacting proteins.

**Interaction motif**	**Binding site on p97**	**Gene name**	**Function**	**References**
UBX	N terminal domain	UBXN1/SAKS1	Negative regulator of ERAD	LaLonde and Bretscher, 2011
		UBXN2A/UBXD4	Unknown	Alexandru et al., 2008
		UBXN2B/p37	Membrane fusion, Golgi and ER biogenesis	Uchiyama et al., 2006
		UBXN2C/p47	Membrane fusion, Golgi and ER biogenesis	Kondo et al., 1997; Yuan et al., 2001; Wang et al., 2004
		UBXN3A/UBXD12/FAF1	Ubiquitin-proteasome pathway	Song et al., 2005
		UBXN3B/UBXD8/ FAF2/ETEA	ERAD, lipid droplets turnover, mRNA stability	Lee et al., 2008, 2010; Mueller et al., 2008; Olzmann et al., 2013; Zhou et al., 2013
		UBXN4/ UBXD2/Erasin	ERAD	Liang et al., 2006
		UBXN6/UBXD1	Endocytosis, turnover of ruptured lysosomes, membrane trafficking	Ritz et al., 2011; Haines et al., 2012; Papadopoulos et al., 2017
		UBXN7/UBXD7	Regulation of transcription factor HIF1α	Alexandru et al., 2008
		UBXN8/UBXD6	ERAD	Madsen et al., 2011
		UBXN9/UBXD9/ASPSCR1	Glucose transpotor trafficking	Bogan et al., 2003
		UBXN10/UBXD3	Ciliogenesis	Raman et al., 2015
		UBXN11/UBXD5	Unknown	By similarity
UBX like	N terminal domain	OTU1/YOD1	DUB, ERAD and clearance of lysosomes	Ernst et al., 2009; Papadopoulos et al., 2017
		Npl4	ERAD, transcription factor maturation	Bays et al., 2001b; Hitchcock et al., 2001; Rape et al., 2001; Ye et al., 2001; Jarosch et al., 2002; Isaacson et al., 2007
		VCPIP/VCIP135	DUB, involved in Golgi reassembly after mitosis, and the formation of transitional endoplasmic reticulum	Uchiyama et al., 2002
PUL	C terminal domain	Ufd3/PLAA	Substrate recruitment in ERAD and mitochondria-associated degradation	Ghislain et al., 1996; Bohm et al., 2011; Wu et al., 2016
PUB	C terminal domain	PNGase	Deglycosylation in ERAD	Li et al., 2006; Zhao et al., 2007
VIM	N terminal domain	VIMP	An ER membrane p97 adaptor in ERAD	Ye Y. H. et al., 2004
		gp78/AMFR	E3 ligase in ERAD	Fang et al., 2001; Zhong et al., 2004
		SVIP	Negative regulation of ERAD	Ballar et al., 2007
		ZFAND2B/AIRAPL	Preemptive quality control of secreted proteins, Signal peptide-mediated translocation, regulation of IGF-1 signaling pathway, tumor suppressor	Braunstein et al., 2015; Osorio et al., 2016; Rahighi et al., 2016
		ANKZF1	A mitochondria p97 adaptor	Heo et al., 2010; Hanzelmann and Schindelin, 2011; Stapf et al., 2011
VBM	N terminal domain	Hrd1/SYVN1	E3 ligase in ERAD	Bordallo et al., 1998; Bays et al., 2001a
		Ataxin-3/MJD/SCA3	DUB, Substrate processing in ERAD	Doss-Pepe et al., 2003; Wang et al., 2006
		UBE4B/Ufd2	E4, Substrate processing in ERAD	Koegl et al., 1999; Mouysset et al., 2006; Bohm et al., 2011
		NUB1/NYREN18	Down-regulator of the NEDD8 conjugation system	Schmidtke et al., 2006
		RHBDD1/RHBDL4	Intramembrane proteolysis, ERAD, apoptosis	Fleig et al., 2012
SHP box	N terminal domain	Ufd1	ERAD, transcription factor maturation	Bays et al., 2001b; Rape et al., 2001; Ye et al., 2001; Jarosch et al., 2002
		Derlin1	ERAD	Lilley and Ploegh, 2004, 2005; Ye Y. et al., 2004
		Derlin2	ERAD	Lilley and Ploegh, 2005; Huang et al., 2013
		SPRTN/DVC1/C1orf124	UV-induced DNA damage response	Davis et al., 2012; Mosbech et al., 2012

Intriguingly, despite the drastic difference in sequence and structure, many p97-interacting motifs, particularly those interacting with the N-domain, bind p97 in a similar mode. Consequently, the binding of many cofactors/adaptors to p97 is mutually exclusive (Meyer et al., 2000; Rumpf and Jentsch, 2006). These observations suggested the existence of distinct populations of p97 complexes in cells, each bearing a different set of partners. Conceptually, the composition of a p97 complex may not be static in cells. Co-factor exchange could occur, which would allow p97 to efficiently switch substrate to meet cellular demands. A similar “adaptor swapping” model has been proposed for the multi-subunit SCF (Skp1, cullin, and F box) ubiquitin ligase, which like p97, uses a collection of adaptors to engage distinct substrates. In this case, adaptor switch is catalyzed by Cand1, a protein exchange factor that stimulates the equilibrium of Cul1-Rbx1 with multiple F box protein-Skp1 modules (Pierce et al., 2013). Whether a similar regulatory strategy exists for p97/Cdc48 remains to be seen. Furthermore, given that the substrate processing cycle is comprised of two mechanistically distinct reactions, namely substrate binding and release, it is conceivable that a regulated hierarchical cofactor binding system may be coupled to ATP hydrolysis to coordinate these processes (Hanzelmann et al., 2011; Meyer et al., 2012).

**Figure 4:**
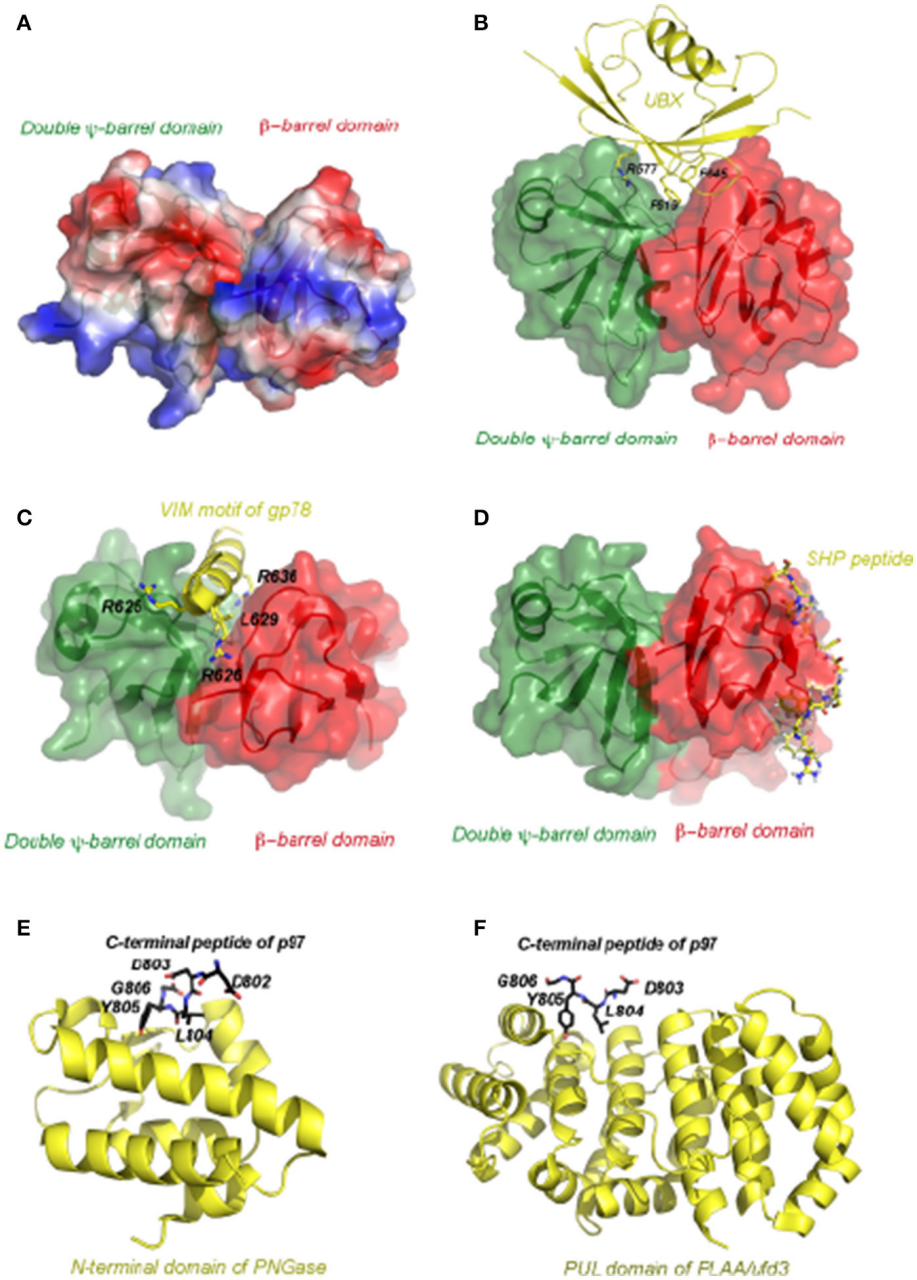
The atomic representation of p97 in complex with various representative interacting motifs. **(A)** The structure of the N-terminal domain of p97 shown as electrostatic potential surface. The positive potential is in blue, negative in red and neutral in white. **(B)** Structure of the p97 N-domain in complex with the UBX domain of FAF1 (PDB:3QC8). The N-domain, depicted as a molecular surface overlaid to a ribbon representation, has the N-terminal double Y-barrel domain colored green and C-terminal β-barrel domain colored red. The UBX domain of FAF1 is depicted as ribbon diagram in yellow. Critical residues for interaction are shown as ball-and-stick models and labeled. **(C)** Structure of the p97 N-domain in complex with the VIM motif of gp78 (PDB:3TIW). Here the VIM motif is shown as helix in yellow and its binding to the N-domain is mostly mediated by charged residues. **(D)** Structure of the p97 N-domain in complex with the Ufd1 derived SHP peptide (PDB:5C1B). Here the SHP peptide is shown as the stick model in yellow and it binds exclusively to the C-terminal β-barrel domain. **(E)** Structure of the N-terminal domain of PNGase in complex with a C-terminal peptide of p97 (PDB:2HPL). The PNGase N-terminal domain is shown in cartoon representation in yellow. The bound peptide is shown as a stick model with five residues (labeled) seen in the structure. The carbon atoms are colored in black, nitrogen in blue and oxygen in red. **(F)** Structure of the PUL domain of FLAA/Ufd3 in complex with a C-terminal peptide of p97 (PDB:3EBB). The PLAA PUL domain is shown in cartoon representation in yellow. The bound peptide is shown as a stick model with four residues visible in the structure. The carbon atoms are colored in black, nitrogen in blue and oxygen in red.

Structural studies have revealed the general principles of p97 complex assembly. To date, one of the best characterized p97 complex is the p47-N-D1 assembly (Dreveny et al., 2004). One crystallographic study showed that the p97 N-domain could be divided into two sub-domains: a N-terminal double Ψ-barrel and a C-terminal β-barrel (Figure 4A). Between the two subdomains features a hydrophobic groove surrounded by patches of charged residues, which is the site bound by the UBX domain found in adaptors such as p47 and FAF1. The interaction usually exploits both hydrophobic and electrostatic forces (Figure 4B). More recently, a collection of structural studies showed that this cleft could be used to engage other p97-binding motifs. For instance, although VIM is unrelated to the UBX domain in both sequence and structure, they both bind to the p97 N-domain at this location (Figure 4C, Hanzelmann and Schindelin, 2011). However, certainly not every N-domain binding protein interacts with p97 in such a manner. An additional surface on the N-domain that binds the SHP box was recently reported (Figure 4D, Hanzelmann and Schindelin, 2016a). Given that some p97 adaptor or adaptor complex contain both UBX and SHP domains (e.g., p47 and the heterodimeric Ufd1-Npl4 complex, Table 1), these adaptors may use a bipartite mechanism to form a complex with p97 (Bruderer et al., 2004; Isaacson et al., 2007; Yeung et al., 2008; Le et al., 2016).

Adaptor/cofactor binding to the C-terminus of p97 has also been studied by crystallography. One such structure is the PUB (PNGase/UBA) domain of the peptide-N-glycanase (PNGase) bound by a 10-residue peptide from the p97 C-terminus dubbed as PUB-interacting motif (PIM) (Figure 4E, Zhao et al., 2007). PNGase is a sugar-processing enzyme responsible for the removal of N-glycan from misfolded glycoproteins retrotranslocated from the ER (Blom et al., 2004). The PUB domain binds PIM in a 1:1 stoichiometry. In this complex, the PIM peptide binds to a conserved surface of the PUB domain (Allen et al., 2006; Zhao et al., 2007). Intriguingly, the conserved residue Y805 in the PIM motif essential for the interaction can be phosphorylated in cells. This post-translational modification may serve a regulatory function in controlling the p97-PNGase interaction (Zhao et al., 2007). Another example is demonstrated by the structure of a complex containing PLAA (phospholipase A2-activating protein) and the C-terminal peptide of p97 (Qiu et al., 2010). PLAA (also named Ufd3 or Doa1) has been implicated in a variety of cellular processes including processing of misfolded mitochondria outer-membrane proteins (Wu et al., 2016), ribophagy (Ossareh-Nazari et al., 2010), endosomal trafficking (Ren et al., 2008; Han et al., 2014), and in regulating the cellular ubiquitin level by an unknown mechanism (Johnson et al., 1995). In the structure, Y805 of p97 is once again located at the binding interface, suggesting that phosphorylation dependent regulation might be a common theme for p97-cofactor interactions (Figure 4F).

Several p97-adaptor assemblies have also been examined by Cryo-EM (Rouiller et al., 2000; Beuron et al., 2006; Pye et al., 2007; Bebeacua et al., 2012). EM studies showed that in the complex of p97 and Ufd1-Npl4 (Pye et al., 2007; Bebeacua et al., 2012), the adaptors bind to both the N- and D1-domain simultaneously. A similar mode of interaction was observed for Fas-associated factor-1 (FAF1) (Ewens et al., 2014).

Whether cofactor binding can cause a conformational change in p97/Cdc48 has not been thoroughly investigated. Structural studies of adaptor-free p97 N-D1 domain (PDB:1E32) or that bound by p47 (PDB:1S3S) or other adaptors showed no obvious change in the structure of p97 upon adaptor binding (Dreveny et al., 2004). However, adaptor-induced conformational changes may only take place in full-length p97 during a normal ATPase cycle, and thus might have escaped detection so far (Isaacson et al., 2007; Zhao et al., 2007; Qiu et al., 2010; Hanzelmann and Schindelin, 2011; Hanzelmann et al., 2011; Kim et al., 2011; Schaeffer et al., 2014). On the other hand, since ATP-dependent conformational changes, particularly those triggered by ATP binding to the D1 domain affect the position of the N-domain, the interaction of p97 adaptors with the N-domain can probably be regulated by the nucleotide state of the D1 ring, as suggested by a recent study (Bulfer et al., 2016).

## Cellular function of p97/Cdc48

Given the substrate diversity, p97 is bestowed a broad function, which has been reviewed extensively (Bug and Meyer, 2012; Dantuma and Hoppe, 2012; Meyer et al., 2012; Yamanaka et al., 2012; Dantuma et al., 2014; Meyer and Weihl, 2014). Due to space constraints, we here only discuss a few relatively better characterized molecular processes, aimed at illustrating the general role of this ATPase in cells.

## Roles in protein homeostasis control

p97/Cdc48 has been implicated in several PQC pathways, and thus is an essential component of the proteostasis regulatory network in eukaryotic cells (Meyer et al., 2012). In general, p97 facilitates the degradation of aberrant proteins by releasing them from cellular structures or large protein complexes. The first identified PQC function for p97 is in ER-associated protein degradation (ERAD), a pathway that eliminates misfolded proteins of the secretory pathway (Smith et al., 2011; Christianson and Ye, 2014; Ruggiano et al., 2014). During ERAD, misfolded proteins are retrotranslocated into the cytosol where they are degraded by the ubiquitin proteasome system. For misfolded luminal proteins, the retrotranslocation process consists of two essential steps. First, a portion of a substrate needs to be moved across the lipid bilayer to enter the cytosol. This reaction is believed to be mediated by a protein retrotranslocation complex containing the multi-spanning membrane ubiquitin ligase Hrd1 (Bordallo et al., 1998; Bays et al., 2001a; Gauss et al., 2006; Carvalho et al., 2010; Stein et al., 2014; Baldridge and Rapoport, 2016). In the second step, p97/Cdc48 is recruited to the site of retrotranslocation via association with proteins present in the retrotranslocation complex. These include Derlins, Hrd1, and VIMP in mammals or Ubxd2 in *S. cerevesiae* (Lilley and Ploegh, 2004; Ye Y. et al., 2004; Neuber et al., 2005; Schuberth and Buchberger, 2005). These proteins each bear a p97 interacting motif, and the interactions with p97 allow it to effectively capture substrates emerging from the retrotranslocation channel (Carvalho et al., 2010). Misfolded proteins then undergo ubiquitination and are dislocated from the membranes by p97 (Bays et al., 2001b; Ye et al., 2001, 2003; Braun et al., 2002; Jarosch et al., 2002; Rabinovich et al., 2002; Flierman et al., 2003; Zhong et al., 2004; Garza et al., 2009). Dislocated ERAD substrates are eventually targeted for degradation by the proteasome (Zhang and Ye, 2014). In addition to ERAD substrates, p97/Cdc48 can also release a few membrane-bound transcription factors without targeting them for degradation (Hitchcock et al., 2001; Rape et al., 2001; Shcherbik and Haines, 2007; Radhakrishnan et al., 2014); instead, these transcription factors are transported into the nucleus to affect gene expression in response to specific stimulating cues.

It has also been demonstrated that p97 can facilitate mitochondria-associated degradation (MAD) by extracting polypeptides from mitochondrial outer membrane (Heo et al., 2010; Xu et al., 2011; Hemion et al., 2014). This process eliminates aberrant polypeptides from mitochondrial outer membrane to maintain mitochondrial protein homeostasis. In addition, regulators of the mitophagy pathway (e.g., mitofusin), which turns over damaged mitochondria can also be subject to degradation by MAD (Tanaka et al., 2010). Upon mitochondrial damage, p97 and Ufd1, Npl4 are recruited to the surface of mitochondria, which is required for clearance of damaged mitochondria by mitophagy (Kimura et al., 2013). The mechanism that recruits p97 to mitochondria in MAD or mitophagy is unclear. One recent study identified a protein named Vms1 (VCP/Cdc48-associated mitochondrial stress-responsive 1) as a potential linker (Heo et al., 2010, 2013), but the role of Vms1 in mitochondria PQC remains controversial (Esaki and Ogura, 2012). In addition, in *S. cerevisiae*, a protein named Doa1 (also named Ufd3) can act in conjunction with Ufd1 and Npl4 to recruit substrates to Cdc48 in MAD (Wu et al., 2016).

Another essential PQC function involving p97 is the degradation of aberrant nascent polypeptides stalled on ribosomes in a process dubbed ribosome-associated degradation (RAD) (Brandman et al., 2012; Defenouillere et al., 2013; Verma et al., 2013). Ribosome stalling occurs when an mRNA in translation is defective (e.g., lack of stop codon, truncated, or damaged in other ways). Such defective mRNAs are rapidly decomposed, but only after they have been “put in test” for fidelity by translation (Brandman and Hegde, 2016). Thus, the execution of this cellular mRNA surveillance program is inevitably associated with the production of aberrant polypeptides, which need to be effectively removed. Using diverse model substrates, it has been demonstrated that a series of factors act in concert to split a stalled ribosome (Pisarev et al., 2010; Shoemaker et al., 2010; Shoemaker and Green, 2011), allowing another ribosome-associated ubiquitin ligase to ubiquitinate aberrant nascent polypeptide (Bengtson and Joazeiro, 2010). Subsequently, a ribosome-associated factor named Rqc1 together with the ubiquitinated substrate recruits p97/Cdc48, which in turn extracts defective polypeptides from the ribosome to promote their degradation by the proteasome (Brandman et al., 2012). Accordingly, inactivation of p97/Cdc48 or its cofactor Ufd1 and Npl4 leads to accumulation of ubiquitinated proteins in complex with the 60S ribosome (Verma et al., 2013).

Several recent studies also implicate p97 and Cdc48 in autophagy, which targets unwanted cellular proteins (including misfolded ones) for lysosomal degradation via autophagasomes. However, the precise function of p97 in this process is controversial, mainly because the substrate(s) regulated by p97 is unclear. Several studies suggest p97 as a positive autophagy regulator because its inhibition causes a phenotype reminiscent of what appears to be an autophagasome maturation defect (Ju et al., 2009; Ju and Weihl, 2010; Bug and Meyer, 2012). In *S. cerevisiae*, a Cdc48 adaptor named Shp1p can bind the autophagy regulator Atg8 to promote macroautophagy (Krick et al., 2010). A more recent study showed that in mammalian cells p97 might be involved in a specialized form of autophagy, which clears ruptured late endosome/lysosome (Papadopoulos et al., 2017). However, another study using a p97 specific inhibitor demonstrated that inhibition of p97 accelerates rather than inhibits autophagasome clearance, increasing the turnover of the autophagy cargo receptor protein p62 (Anderson et al., 2015). This suggests an inhibitory role for p97 in autophagy. Additional studies are required to clarify the precise role of p97 in autophagy.

Other than the proposed “segregase” activity, p97 may also act as a chaperone to transport misfolded polypeptides to the proteasome for degradation, or to simply prevent protein aggregation (Yamanaka et al., 2004; Nishikori et al., 2008; Gallagher et al., 2014; Neal et al., 2017). This activity might be critical for degradation of aggregation-prone nuclear proteins in budding yeast (Gallagher et al., 2014). Additionally, p97 was also shown to facilitate the clearance of non-translating messenger ribonucleoprotein complexes from stress granules via an unknown mechanism (Buchan et al., 2013). Other misfolded proteins that are potential p97 substrate include misfolded unassembled cytosolic and nuclear proteins (Xu et al., 2016). Lastly, in addition to acting directly on misfolded proteins, p97 can also control the stability of certain stress regulators. For example, the complex of p97 and UbxD7 was shown to work with a SCF ubiquitin ligase to target hypoxia-inducible factor 1 alpha (HIF1α) for degradation (Alexandru et al., 2008). More recently, it was shown that p97 could also control the glutamine-regulated turnover of glutamine synthetase as well as the half-life of several cullin-ring ubiquitin ligase substrates (Nguyen et al., 2017; Tao et al., 2017).

## Other functions

By releasing polypeptides from the chromatin in a manner analogous to that in ERAD, p97 and Cdc48 can function in an array of nuclear processes known as chromatin-associated degradation (Dantuma et al., 2014). Many nuclear p97 substrates have been identified. These include RNA polymerase (Pol) II complex (Verma et al., 2011), transcriptional repressor α2 (Wilcox and Laney, 2009), and CMG DNA helicase (Maric et al., 2014) in budding yeast, and the DNA replicating licensing factor CDT1 (Franz et al., 2011; Raman et al., 2011), replisome component Mcm7 (Moreno et al., 2014), DNA repairing proteins DDB2, XPC, and Rad52 (Bergink et al., 2013; Puumalainen et al., 2014), mitosis regulator Aurora B kinase (Ramadan et al., 2007; Sasagawa et al., 2012), certain DNA polymerases (Davis et al., 2012; Mosbech et al., 2012), the DNA double strain break (DSB) repair protein Ku70/80 (van den Boom et al., 2016), the RNA binding protein HuR (Zhou et al., 2013), and the polycomb protein L3MBTL1 (Acs et al., 2011) in metazoa. These substrates link p97 to various nuclear pathways ranging from gene expression control to DNA damage response. Intriguingly, although most of these proteins have been shown to undergo ubiquitination in cells, not all of them are subject to proteasome-mediated degradation.

In mitotic cells, p97/Cdc48 can regulate vesicle fusion at the exit of mitosis when the Golgi apparatus and the ER network need to be re-shaped (Kondo et al., 1997; Rabouille et al., 1998; Kano et al., 2005b,a; Uchiyama and Kondo, 2005). This process involves two adaptors p47 (Kondo et al., 1997; Meyer et al., 2002) and p37 (Uchiyama et al., 2006). In addition, a p97-associated deubiquitinase named VCIP135 is required (Uchiyama et al., 2002). It has been proposed that p97 may act on Syntaxin 5 to regulate vesicle fusion (Rabouille et al., 1998; Roy et al., 2000). In post-mitotic cells such as neurons, the complex of p97-p47 has been implicated in maintaining the tubular ER structure in order to control protein synthesis (Shih and Hsueh, 2016).

Several lines of evidence suggested that mammalian p97 might also regulate receptor-mediated endocytosis (Bug and Meyer, 2012; Kirchner et al., 2013). Proteomic studies uncovered the early endosome-associated antigen 1 (EEA1) and Clathrin as p97-interacting proteins (Pleasure et al., 1993; Ramanathan and Ye, 2012). Functionally, inhibition of p97 delays lysosomal targeting of an endocytosis cargo. p97 inhibition also causes clustered and enlarged early endosomes, which might result from increased EEA1 oligomerization and thus uncontrolled endosome tethering and fusion (Ramanathan and Ye, 2012). In another study, the plasma membrane protein caveolin was found to interact with p97 and UbxD1. In p97-deficient cells, enlargement of endosome was similarly observed, and the trafficking of caveolin to late endosomes is affected (Ritz et al., 2011). The precise function of p97 in endocytosis remains to be elucidated, but it might be mechanistically related to the proposed function of p97 in autophagy.

In addition to vesicular trafficking, p97 may also control protein transport in a non-vesicular manner as it was recently demonstrated that the complex of p97 and UBXN10 mediates protein transport into cilia to control ciliogenesis (Raman et al., 2015). Mammalian p97 has also been shown to regulate NFκB signaling by controlling the stability of the small inhibitory protein IκB in the canonical NFκB pathway (Dai et al., 1998; Li et al., 2014) or by facilitating the processing of the p100 subunit in the alternative NFκB activation pathway (Zhang Z. et al., 2015). The p97 was also shown to regulate the stability of RIG-1, a viral RNA sensor in innate immunity (Hao et al., 2015) as well as the activity of adipose triglyceride lipase (ATGL), an enzyme that controls lipid droplet biogenesis (Olzmann et al., 2013).

## Relevance to human disease

Genetic studies in the past decade have linked a collection of p97 mutations to human diseases including MSP1 (multisystem proteinopathy 1) [also named IBMPFD (Inclusion Body Myopathy associated with Paget's disease of the bone and Frontotemporal Dementia)], FALS (familial amyotrophic lateral sclerosis), CMT2Y (Charcot-Marie-Tooth disease, type 2Y) (Dyck and Lambert, 1968; Watts et al., 2004; Johnson et al., 2010; Abramzon et al., 2012; Bucelli et al., 2015), hereditary spastic paraplegias (HSP), Parkinson's disease (PD), and Alzheimer's disease (AD). Mechanistic studies suggest that a major dysfunction of p97 in association with these disease conditions is deregulation of the proteostasis network.

## Multisystem proteinopathy 1 (MSP1)

MSP1/IBMPFD is a severe autosomal dominant disorder. Patients experience progressive tissue damages in either the muscles (myopathy), the bones (Paget's disease of the bone, PDB), and/or the brain (frontotemporal dementia, FTD). To date, more than 40 mutations covering 29 different positions in p97 have been reported in MSP1/IBMPFD patients (Nalbandian et al., 2011; Mehta et al., 2013). However, as patients bearing the same mutation from a single family can show drastically different symptoms with differing on-set ages, other genetic or environmental factors may also make significant contribution to the disease etiology.

At the cellular level, muscle fibers from MSP1/IBMPFD patients often contain vacuoles that are stained by antibodies against ubiquitin and p97 (Watts et al., 2004). In brain tissues, nuclear inclusions containing ubiquitin and p97 were also frequently detected in neurons (Kimonis and Watts, 2005). More recent studies also found TAR DNA-binding Protein-43 (TDP-43) accumulating in patient tissues (Weihl et al., 2008). Genetic interactions between TDP-43 and p97 have also been revealed, which may regulate subcellular distribution of TDP-43 (Ritson et al., 2010). These findings suggested a role of p97 in controlling the neurotoxicity of aggregation-prone misfolded polypeptides, possibly by regulating their stability, solubility, or subcellular localization.

**Figure 5:**
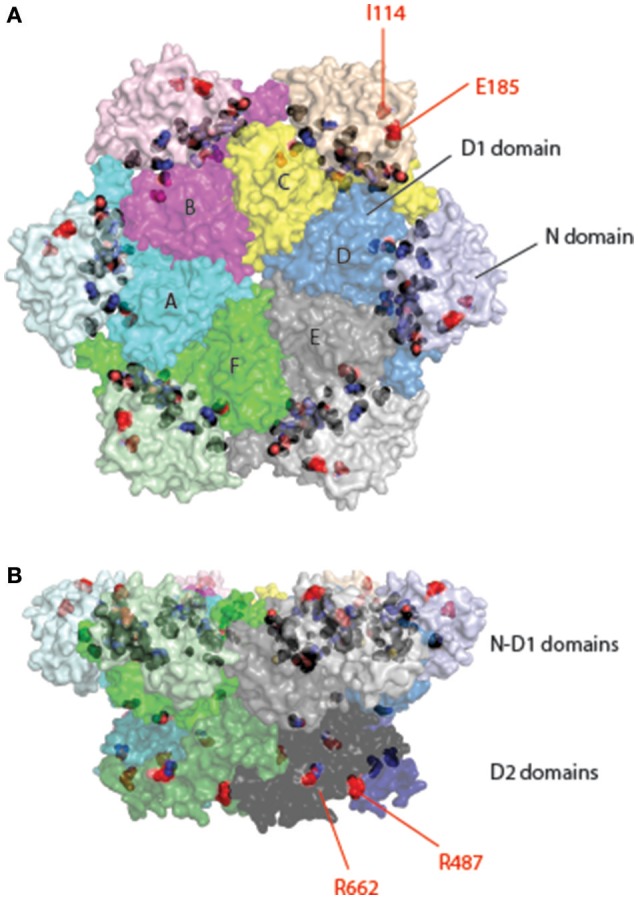
Location of pathogenic mutations in the structure of full-length p97. Surface representation of the structure of p97 is decorated with pathogenic mutations in p97 identified from patients of various muscular and neurological disorders. Each subunit is given a unique color with distinct shades for different domains. Interface mutations are colored in black and non-interface mutations in red and labeled. The non-interfacial mutations are mostly identified in ALS patients. **(A)** Top view. **(B)** Side view.

Structural studies revealed that MSP1/IBMPFD mutations are mostly mapped to or near the interface between the N and D1 domains of p97 (Figure 5). Because patients carrying a single allele of any MSP1 mutations develop normally, these mutations apparently only cause non-optimal performance in p97 ATP hydrolysis cycle, accumulating damages to p97-dependent cellular processes that culminate in neuronal cell death in adulthood (Kimonis et al., 2000). These mutations could affect the function of p97 in multiple facets. For example, many mutations appear to weaken the affinity of the D1 domain for ADP (Tang et al., 2010), resulting in increased (2–4-fold) D2 ATPase activity and a loss in coordinated N-domain movement (Weihl et al., 2006; Halawani et al., 2009; Tang et al., 2010; Tang and Xia, 2013; Schuetz and Kay, 2016). Moreover, while some cofactors can elevate or inhibit the ATPase activity of wild-type p97, these regulations do not seem to occur with certain disease-associated mutants (Zhang X. et al., 2015). These observations collectively suggest that mutation-induced structural instabilities might have caused a loss in the fine-tuned ATPase cycle, causing cell damages. In addition, biochemical studies also demonstrated an effect of certain mutations on cofactor association (Fernandez-Saiz and Buchberger, 2010; Tang and Xia, 2016b), whereas in the case of p37 and p47, nucleotide dependent regulation of cofactor binding appears to be abolished with disease-associated mutants (Bulfer et al., 2016). *In vivo*, subtle deregulation of p97 ATPase activity might result in a gain-of-function phenotype in sensitive tissues, as demonstrated recently by a study using a *Drosophila* IBMPFD model (Zhang et al., 2017). Consistent with this view, Blythe and colleagues show that an IBMPFD mutant that has a moderately increased ATPase activity and can unfold ubiquitinated GFP more efficiently than wild-type p97 (Blythe et al., 2017).

## Familiar amyotrophic sclerosis (FALS)

Autosomal dominantly inherited amyotrophic lateral sclerosis (ALS) (also known as Lou Gehrig's disease) is a progressive neurodegenerative disease. It mainly affects the motor neurons in the brain and spinal cord, resulting in death from respiratory failure. While most ALS cases were caused by sporadic mutations, about 10% are considered “familial” because often more than one individual in a family develops the disease. Mutations in at least 18 genes have been identified in familial ALS. Among them, p97 mutations account for less than 2% (Johnson et al., 2010; Koppers et al., 2012; Kwok et al., 2015). There are 18 reported mutations appearing in 12 different positions. Although there is a significant overlap between MSP1 and familial ALS mutations, mutations linked to familial ALS can be found in the D2 domain and many of them are not located at the interface between the N and D1 domains (e.g., I114V in the N domain, R487H, and R662C in the D2 domain) (Figure 5). How these mutations alter the function of p97 remains unclear. However, as the pathological hallmark of the disease, loss of motor neurons, is often linked to the appearance of ubiquitin-positive inclusions and/or deposition of TDP-43-positive aggregates (Johnson et al., 2010), ALS pathology may be at least in part attributed to defects in cellular protein homeostasis.

## Charcot-marie-tooth disease, type 2Y (CMT2Y)

Charcot-Marie-Tooth disease (CMT) is an autosomal dominant axonal peripheral neuropathy characterized by distal muscle weakness and atrophy associated with length-dependent sensory loss. Like ALS, CMT is a clinically and genetically heterogeneous disorder and is divided into subtypes based on genetics, pathology, and electrophysiology of the disease (Dyck and Lambert, 1968). Missense mutations in p97 were recently identified in patients of the CMT2 Y-subtype (Gonzalez et al., 2014; Jerath et al., 2015). As most patients with CMT2Y do not obtain a genetic diagnosis, the number of cases bearing mutations in p97 should be higher than expected. Intriguingly, in addition to p97, other CMT2-associated genes identified include chaperones such as Hsp27 and Hsp22 (Houlden et al., 2008; Nakhro et al., 2013). These observations once again link the etiology of this disease to deregulation of the proteostasis network.

## p97 as a potential anti-cancer target

**Figure 6:**
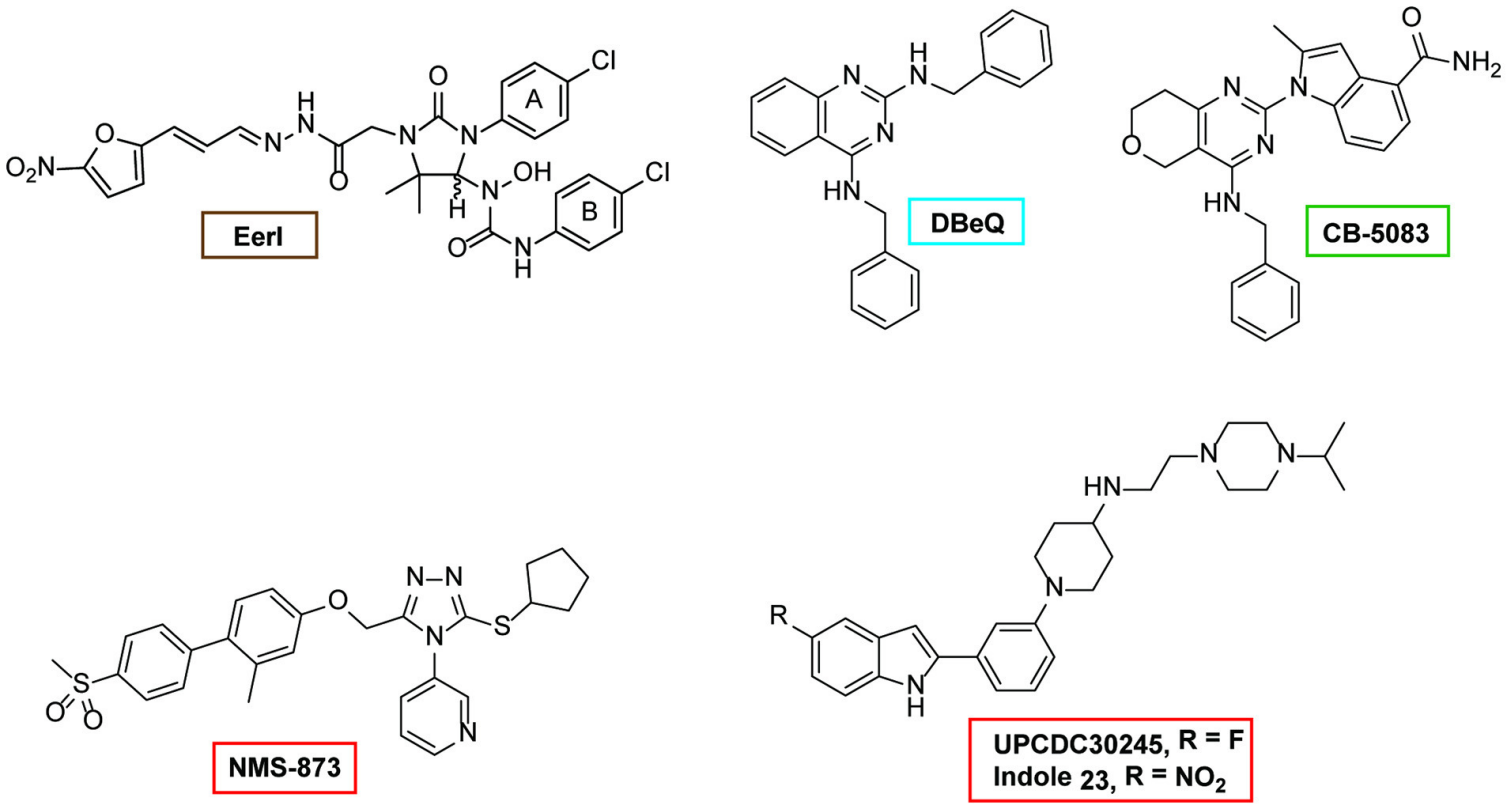
Structures of selected p97 inhibitors. Shown are the chemical structure of some well characterized p97 inhibitors. EerI inhibits p97 function by binding to its D1 domain. DBeQ was the first reversible inhibitor that blocks the D2 ATPase activity. CB-5083 is a derivative of DBeQ, but it is much more potent than DBeQ. CB-5083 and NMS-873 are the most potent and specific p97 inhibitor identified to date. UPCDC30245 is a recently identified inhibitor and its binding to p97 has been characterized by EM.

Given the important roles played by p97 in diverse cellular processes, specific inhibitors of p97 can be useful tools for dissecting the mechanism of p97 action. Early chemical screens focusing on compounds that inhibit ERAD identified two structurally related chemicals (Fiebiger et al., 2004). Characterization of these compounds led to the discovery of the first p97 inhibitor-Eeyarestatin (EerI) (Figure 6) (Wang et al., 2008, 2010). Intriguingly, although EerI binding causes a conformational change in p97, it does not seem to affect nucleotide hydrolysis by the D2 domain. Whether it affects ATP hydrolysis by D1 is unclear, nor is the inhibitory mechanism by EerI (Wang et al., 2010). Nevertheless, in tissue culture cells, EerI induces several key phenotypes attributed to p97 inhibition such as the accumulation of polyubiquitinated proteins, ERAD inhibition, ER stress induction, and apoptosis (Wang et al., 2009). Importantly, EerI has significant cancer-killing activities *in vitro* as it preferentially kills cancer cells isolated from patients; and it can synergize with the proteasome inhibitor Bortezomib to induce apoptosis in cancer cells (Wang et al., 2009). These observations provide a rationale for targeting p97 as a new anti-cancer therapy.

More recently, chemical screens in search of compounds directly targeting p97 have been conducted. Chou and colleagues reported the first reversible p97 D2 inhibitor, DBeQ (Chou et al., 2011). Subsequent work has optimized this chemical, leading to a collection of more potent and specific p97 inhibitors (Chou et al., 2013, 2014; Chapman et al., 2015; Zhou et al., 2015). An independent effort from Magnaghi and colleagues identified several competitive and non-competitive inhibitors that also target the D2 domain (Magnaghi et al., 2013). These p97 D2 inhibitors are highly specific and potent (Magnaghi et al., 2013; Anderson et al., 2015). Structural modeling and Cryo-EM studies have revealed the potential inhibitory mechanism of one p97 inhibitor, the small allosteric inhibitor UPCDC30254 observed at the interface between the D1 and D2 domains, seems to prevent the propagation of conformational changes necessary for p97 function (Banerjee et al., 2016). Treatment of human cancer cell lines with these allosteric inhibitors confirmed that inhibition of p97 indeed induces cell death in different cancer cell lines (Chou et al., 2011, 2013; Magnaghi et al., 2013; Anderson et al., 2015). Along this line, it is noteworthy that a reversible p97 inhibitor named CB-5083 has produced promising anti-cancer effects in mouse xenograft tumor models and is now being evaluated in clinical trials (Anderson et al., 2015; Zhou et al., 2015). Lastly, the use of these inhibitors in basic research has started to reveal novel p97 functions in DNA repair, turnover of ruptured lysosomes etc. (van den Boom et al., 2016; Papadopoulos et al., 2017).

In addition to the above-mentioned inhibitors, efforts from several groups have resulted in a large collection of p97 inhibitors (Figure 6) (Yi et al., 2012; Polucci et al., 2013; Cervi et al., 2014; Kang et al., 2014; Alverez et al., 2015; Chapman et al., 2015; Tao et al., 2015; Ding et al., 2016; Gui et al., 2016). Among them, it is particularly worth mentioning that several are natural products. Although these chemicals are not thoroughly characterized and their potency is often limited, research along this direction may lead to a safer p97 inhibitor better suited for cancer therapy.

## Conclusion remarks and perspective

Through years of studies, we have accumulated a large body of knowledge on the structure and function of p97/Cdc48. Specifically, the identification of new p97 cofactors and substrates has revealed a whole new set of biological functions for this essential chaperone system, and it is anticipated that future studies will further expand the p97 functional repertoire. By contrast, mechanistic dissection of the molecular nature of the “segregase” activity has lagged behind, and many fundamental questions remain unresolved. Among them, the most intriguing one is how conformational changes in p97 generate the proposed “segregase” activity. The recently developed *in vitro* GFP unfolding assay represent a major step toward fully elucidating the mechanism of this important enzyme. Another key question is to understand the hierarchical organization of cofactor binding in the context of the ATPase cycle and substrate binding cycle. Moreover, animal models bearing disease-associated mutations are needed in order to better appreciate the connections between p97 dysfunction and human diseases. The recent advance in CRISPR technology should dramatically ease the development of these animal models. Finally, given the promising anti-cancer effect of p97 inhibitors, it is anticipated that more p97 inhibitors will be sought, and studies in this direction may one day produce a new class of anti-cancer agent.

## Author contributions

TZ and WT prepared the figures and tables, prepare part of the manuscript. DX and YY wrote the manuscript.

### Conflict of interest statement

The authors declare that the research was conducted in the absence of any commercial or financial relationships that could be construed as a potential conflict of interest.
